# Tissue multifractality and Born approximation in analysis of light scattering: a novel approach for precancers detection

**DOI:** 10.1038/srep06129

**Published:** 2014-08-20

**Authors:** Nandan Das, Subhasri Chatterjee, Satish Kumar, Asima Pradhan, Prasanta Panigrahi, I. Alex Vitkin, Nirmalya Ghosh

**Affiliations:** 1 Dept. of Physical Sciences, IISER- Kolkata, Mohanpur 741 252, Nadia, West Bengal, India; 2 Department of Physics, IIT Kanpur, Kanpur – 208016, India; 3 Department of Medical Biophysics and Radiation Oncology, University of Toronto, Canada M5G 2M9; 4 These authors contributed equally to this work.

## Abstract

Multifractal, a special class of complex self-affine processes, are under recent intensive investigations because of their fundamental nature and potential applications in diverse physical systems. Here, we report on a novel light scattering-based *inverse* method for extraction/quantification of multifractality in the spatial distribution of refractive index of biological tissues. The method is based on Fourier domain pre-processing via the Born approximation, followed by the Multifractal Detrended Fluctuation Analysis. The approach is experimentally validated in synthetic multifractal scattering phantoms, and tested on biopsy tissue slices. The derived multifractal properties appear sensitive in detecting cervical precancerous alterations through an increase of multifractality with pathology progression, demonstrating the potential of the developed methodology for novel *precancer* biomarker identification and tissue diagnostic tool. The novel ability to delineate the multifractal optical properties from light scattering signals may also prove useful for characterizing a wide variety of complex scattering media of non-biological origin.

Studies on structures and processes that exhibit self-similarity have evoked intensive investigations recently because of their fundamental nature and potential applications in diverse areas ranging from complex biological systems, material structures, electrical circuits to various optical phenomena^1^,^2^,^3^. Fractal measures are characterized by regular (exact fractals) or random (statistical fractals) hierarchical scaling down to arbitrarily small scales^1^,^2^,^3^. While most of the aforementioned self-similar processes typically exhibit monofractal behaviour that can be adequately described by a single scaling exponent, a few special class of complex processes are associated with more complicated scaling behaviour which may be thought of as consisting of many interwoven fractal subsets, each of them characterized by their own local scaling exponent^4^. Such complex self-affine processes are also ubiquitous in nature, and are extensively studied for diverse applications, for example in physiological time series of heartbeat^5^,^6^, turbulence^7^,^8^,^9^, Sun's magnetic field dynamics^10^, stock market fluctuations^11^ and so forth.

Elastically scattered light contains information about the spatial frequency spectrum of the investigated object, providing a useful route to probe the self-similarity in the spatial distribution of its refractive index (RI). Indeed, such light scattering measurements have been explored for characterizing the fractal structures of biological tissues^12^,^13^. Elastic scattering of light in tissue originates from the micro/nano-scale spatial fluctuations of RI at the cellular, sub-cellular and extra-cellular levels^14^. Accordingly, subtle changes in the tissue physical/optical properties leave its characteristic signatures, as manifest in the variation of scattered light intensity (either as a function of angle or wavelength). We have recently shown that the spatial distribution of tissue refractive index exhibits multifractality, indicative of its morphological and ultra-structural tissue content^15^. Extraction and quantification of these multifractal optical properties from light scattering signal may prove to be valuable for non-invasive detection of diseases, including early detection of cancer or even precancer. We thus address the outstanding challenge of the corresponding *inverse* problem; that is, given a tissue light scattering signal (either as a function of angle or wavelength), is it possible to extract its multifractal properties? In tissues, the wide range of spatial scaling of the inhomogeneities and the complicated nature of the spatial correlations present between them contributes in a complex intertwined way to the light scattering signal. The method we propose is based on Fourier domain pre-processing via the Born approximation, followed by the Multifractal Detrended Fluctuation Analysis. The approach is experimentally validated in synthetic multifractal scattering phantoms, and tested on tissue slices (tissue sections of human cervix of different pathology grades). The light scattering-derived *multifractal tissue optical properties* yield valuable information on subtle changes in the index inhomogeneity distribution of tissue; they also appear sensitive in detecting cervical precancerous alterations through an increase of multifractality with pathology progression. The potential of the developed methodology for novel *precancer* biomarker identification as a tissue diagnostic tool is thus suggested.

## Theory

### Multifractal Detrended Fluctuation Analysis (MFDFA)

A statistically homogeneous fractal (monofractal) series is one whose variance exhibits a power law scaling described by a single global Hurst exponent, *H* (0 < *H* < 1)^1^,^2^,^3^. A multifractal signal, on the other hand, exhibits many interwoven fractal subsets characterized by different local Hurst exponents^4^,^5^. Multifractal Detrended Fluctuation Analysis (MFDFA) is a generalized approach to characterize such complex multi-affine processes^4^,^16^. Briefly, the fluctuation profile *Y*(*i*) (series of length N, *i* = 1…..*N*) is divided into N_s_ = int (N/s) non-overlapping segments *m* of equal length s. The local trend of the series (*y_m_*(*i*)) is determined by least square polynomial fitting of each segment *m* of the series. These are then subtracted from the segmented profile to yield the detrended fluctuations, and the resulting variance is determined as^16^

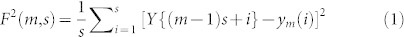
The generalized moment (*q*) dependent fluctuation function is then constructed by averaging the variances over all the segments as 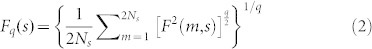
The scaling behavior is subsequently studied under the assumption of power law scaling F_q_(s) ~ s^h(q)^. Using this approach, any non-stationary multifractal signal can be characterized via two sets of scaling exponents: (i) the generalized Hurst exponent *h*(*q*), and (ii) the classical multifractal scaling exponent *τ*(*q*), along with the singularity spectrum *f*(*α*). These are related as^16^

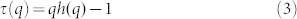

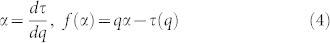


Where *α* is the singularity strength and the full width of *f*(*α*); Δ*α* (taken at *f*(*α*) = 0) is a quantitative measure of multifractality.

### Light scattering based inverse analysis in Born approximation

The MFDFA approach can be directly employed on spatial variation of refractive index to quantify signature of multifractality from any random medium such as tissue^15^. Here, we describe an *inverse* analysis strategy for extraction and quantification of multifractality from the corresponding light scattering signal. Consider a weakly fluctuating scattering medium having normalized RI fluctuations 
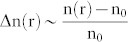
. Here, r is the location within the volume and n_0_ is the average RI of the medium; the fluctuating part of index Δn(r) gives rise to phase distortion and scattering. In the first order Born approximation (weak variations of Δn(r)), the elastic scattering field for scalar excitation can be related to Δn(r) via a Fourier transformation^17^,^18^. For continuous random media such as tissues, we further assume that the index variations arise from statistical inhomogeneities having different spatial dimensions, albeit with similar amplitude of fluctuating index δn (scatterers having different sizes but similar RI)^18^,^19^,^20^. With this approximation, the expression for scattered intensity is given by 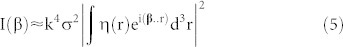
Here, k = 2π/λ, **β** is the scattering vector with modulus β = 2k sin(θ/2), θ is the scattering angle, λ is the wavelength (β is related to the spatial frequency *ν* via β = 2πν); *σ* = n_0_*δn* represents the index fluctuation strength and η(r) is the corresponding spatial distribution of index inhomogeneities. It is evident that any signature of multifractality hidden in η(r) would be encoded in I(β), in the Fourier domain. Consequently, for media exhibiting statistical self-similarity in index fluctuations, the information on multifractality can in principle be obtained from the scattering signal as 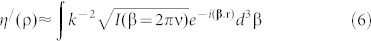
The parameter *η*^/^(ρ) can be interpreted as index inhomogeneity distribution with spatial scale ρ, representing the randomness of the mediumin statistical sense, ρ = |*r* − *r*^/^| is the distance between any two medium points. In what follows, we demonstrate that *η*^/^(ρ) contains the essential multifractal features of index fluctuations in complex systems such as tissues, which can be extracted by employing multifractal analysis. For this purpose, *(i)* we employ MFDFA directly on the spatial variation of tissue RI, obtained from differential interference contrast (DIC) images of human cervical pathology (*forward* analysis); and *(ii)* quantify the multifractal tissue optical properties by employing MFDFA on the Fourier pre-processed light scattering data *η*^/^(ρ) (*inverse* analysis).

## Results and discussion

### Evidence of multifractality in tissue refractive index fluctuations

**Figure 1:**
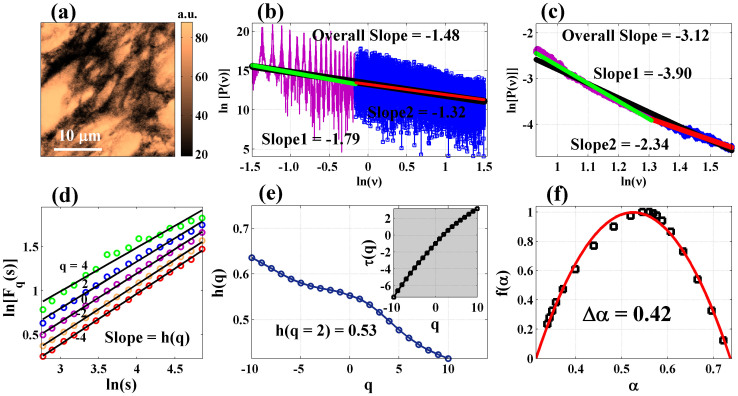
Evidence of multifractality in the spatial variation of tissue RI. (a) DIC image of typical Grade I precancerous human cervix (connective tissue slice). (b) Fourier power spectrum of the generated 1D RI fluctuation series (shown in natural logarithm scale). Fitting at two selected ν-ranges (lower (green) and higher (red), respectively) and overall fitting (black) yield different values for slope (manifestation of multifractality) (c) The light scattering signal [P(ν) = I(ν) × k^−4^] recorded from the same tissue also yields multiple spectral slope exponents (fitting at two selected ν ranges and overall fitting on the entire ν range are shown). (d), (e), (f): Results of the MFDFA *forward* analysis on the detrended (by least square polynomial fitting) spatial index fluctuations (from the DIC image). (d) The variation of the moment (*q*) dependent fluctuation function *F_q_*(*s*) with length scale *s* (constructed using Eq. 2). The points represent the MFDFA-derived values of *F_q_*(*s*) and the lines are power law fitting. Considerable variation in the slopes of log *F_q_*(*s*) *vs* log *s* with varying q confirms multifractality. (e) The variation of the generalized Hurst exponent *h*(*q*) (derived from the slopes of log *F_q_*(*s*) *vs* log *s*) and the classical scaling exponent *τ*(*q*) (derived using Eq. 3) (inset). The points represent the MFDFA-derived values and the lines are guide for the eye (for these and all subsequent figures displaying these parameters). (f) The resulting singularity spectrum *f(α)* (derived using Eq. 4). The points represent the derived values for *f(α)* and the line represents the fitted data (using 2^nd^ order polynomial) for this and all subsequent figures displaying *f(α)*. The value for the full width of the fitted singularity spectrum, *Δα* (taken at *f(α)* = 0) is noted in the figure.

The evidence of multifractality in spatial variation of tissue RI is presented in Figure 1, using the DIC image of the connective tissue regions of typical grade-1 dysplastic/precancerous human cervical tissue. The recorded DIC image (Figure 1a) was pixel-wise unfolded in one linear direction to obtain the representative one dimensional index fluctuation series. This 1D RI fluctuation series was then analyzed via the Fourier and MFDFA methods. The resulting power spectrum of Fourier transformation (P(ν), ν being the spatial frequency here in μm^−1^) is shown in Figure 1b (axes are shown in natural logarithm scale). Statistical self-similarity is manifested as a power-law scaling of the spatial frequency distribution (*P*(*ν*) ≈ *ν*^−*γ*^) (Fig. 1b). As seen, the power law coefficient (slope *γ*) is not uniform throughout the entire ν range (Fig. 1b), suggesting multifractality. As expected from the Born approximation (equation (5)), the light scattering [P(ν) = I(ν) × k^−4^, 
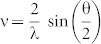
, θ = 150°, λ = 400–760 nm shown here] signal also yields multiple values for the spectral exponent (Fig. 1c). A factor of ~2 differences in the slope scaling exponents between the two approaches is due to the former being obtained from 1D Fourier transform of the index fluctuation, while the latter emerges via a 3D Fourier analysis of the scattering potential.

The corresponding results of MFDFA *forward* analysis are shown in Fig. 1d, e and f. Considerable variations in the slopes of log*F_q_*(*s*) *vs* log*s* with varying moment *q* furnish supporting evidence of multifractality (Fig. 1d). Its strength is subsequently quantified via the moment-dependent generalized Hurst exponent *h*(*q*) (Fig. 1e, derived from the slopes of log*F_q_*(*s*) *vs* log*s*) and the width (Δ*α*) of the singularity spectrum *f*(*α*) (Fig. 1f, derived using equation (4)). Significant variations in *h*(*q*) and considerable magnitude of *Δα* ( = 0.42) demonstrate strong multifractality in the spatial variations of tissue RI.

### *Inverse* analysis of multifractality from light scattering signal

**Figure 2:**
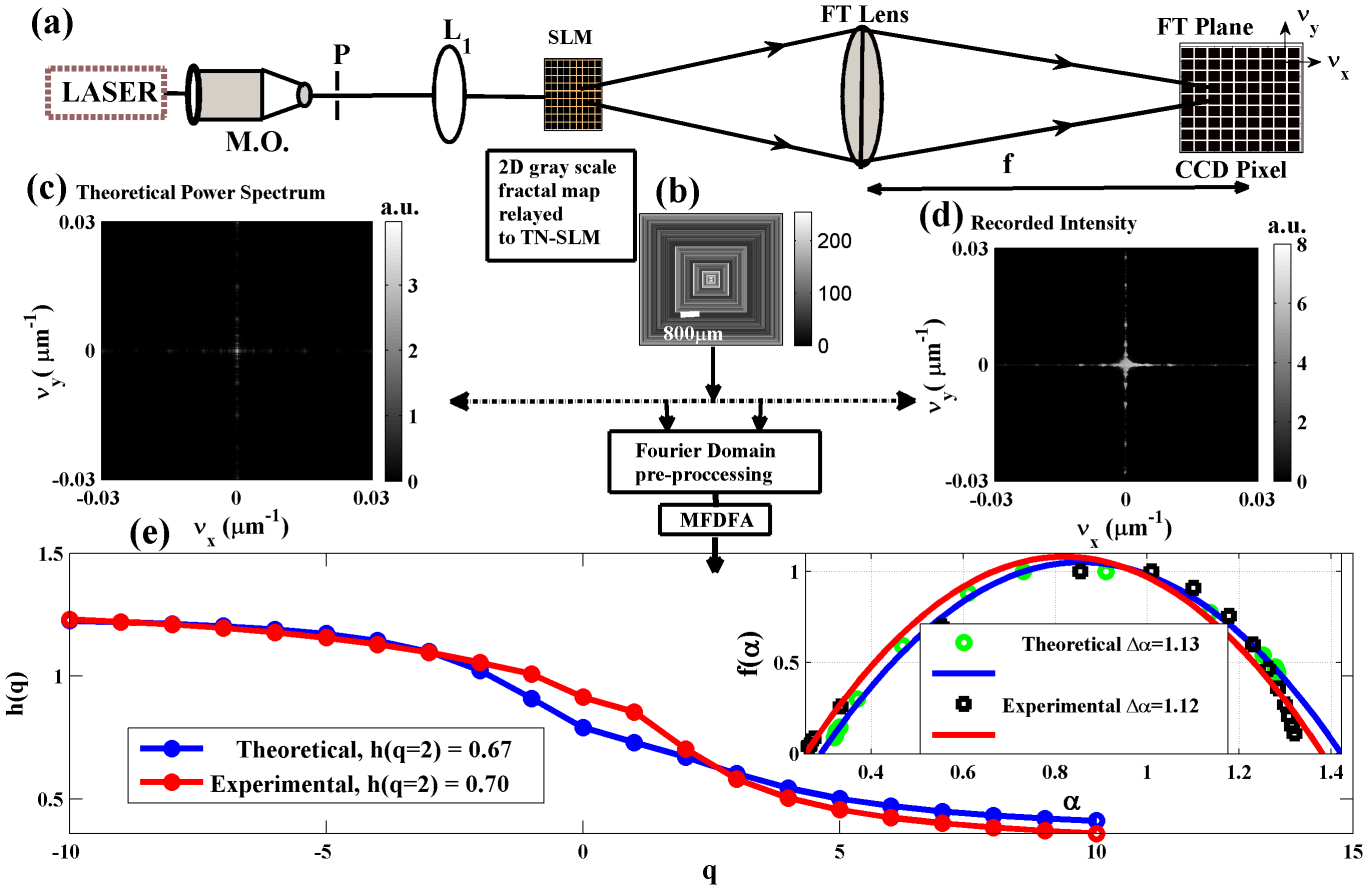
Control validation experiments on synthetic statistical multifractal scattering object. (a) Fourier optics experimental arrangement. MO: microscope objective; P: pinhole; L_1_: collimating lens; FT Lens: Fourier transforming lens (focal length *f*). (b) Multifractal object realized by modulating the pixels of a *transmissive* liquid crystal-based twisted nematic spatial light modulator (SLM) through binomial multifractal model (input *h*(*q* = *2*) * = * 0.69). Color bar represents grey level values (0–255). (c) Theoretical Fourier power spectrum of the multifractal image. (d) The experimentally recorded intensity in the CCD (Fourier) plane, shown as spatial frequency 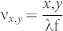 (color bars represent intensity value). Fourier domain pre-processing was performed on both sets of data (c) and (d), which were subsequently analyzed using MFDFA. (e) The MFDFA-derived generalized Hurst exponent *h(q)* for the theoretical (blue) and experimental (red) data. The inset shows corresponding singularity spectra *f(α)*. The values for the width of *f(α)*, (Δα), are also noted in the inset. Excellent agreement between the experimentally derived value *h*(*q* = *2*) * = * 0.70, obtained from the theoretical data (*h*(*q* = *2*) = 0.67) and the controlled input for the synthesized fluctuation series (*h*(*q* = *2*) * = * 0.69), suggests self-consistency in *inverse* analysis of multifractality.

We now turn to the more challenging *inverse* problem of extracting the multifractal optical properties from the light scattering signal. We employ MFDFA on the Fourier pre-processed light scattering data *η*^/^(ρ)(equation (6)). While the Born approximation for modelling light scattering in tissues is widely used^12^,^13^,^14^,^18^,^19^,^20^,^21^,^22^, employing MFDFA on the Fourier pre-processed light scattering signal is novel and as such requires validation. Making use of the Fourier optics experimental arrangement (see Figure 2a and Methods), validation was performed on synthetic statistical mono/multifractal scattering (diffracting) objects whose self-similar properties were user controlled. The mono/multifractal objects were realized by modulating the grey levels of the pixels of a *transmissive* liquid crystal-based twisted nematic spatial light modulator (SLM) through fluctuation series synthesized either by fractional Brownian motion (monofractal) or binomial multifractal model^2^,^16^ (see Methods and Supplementary information). Figure 2 shows the grey level distributions in the SLM pixels for a multifractal object (Fig. 2b), theoretical Fourier power spectrum of the synthesized multifractal image (Fig. 2c), corresponding variation of the recorded intensity in the CCD (Fourier) plane (Fig. 2d), and the results of MFDFA analysis on the Fourier pre-processed theoretical power spectrum and experimental intensity data (Fig. 2e). Comparison of *h(q)* spectrum derived from the experimental data and from the theoretical one shows excellent agreement. The experimental value *h*(*q* = *2*) * = * 0.70, that obtained from the theoretical data (*h*(*q* = *2*) * = * 0.67) and the known input for the fluctuation series (*h*(*q* *=* *2*) * = * 0.69), are in close agreement (note, *h*(*q* = *2*) correspond to the Hurst exponent of an equivalent monofractal series^2^,^16^). Based on this and other continuing validation studies (see Supplementary information), the strategy of Fourier domain preprocessing and its subsequent analysis using MFDFA appears valid for *inverse* extraction of multifractality, and may be relevant for tissue examinations.

### Multifractal tissue optical parameters as novel biomarkers for precancer detection

**Figure 3:**
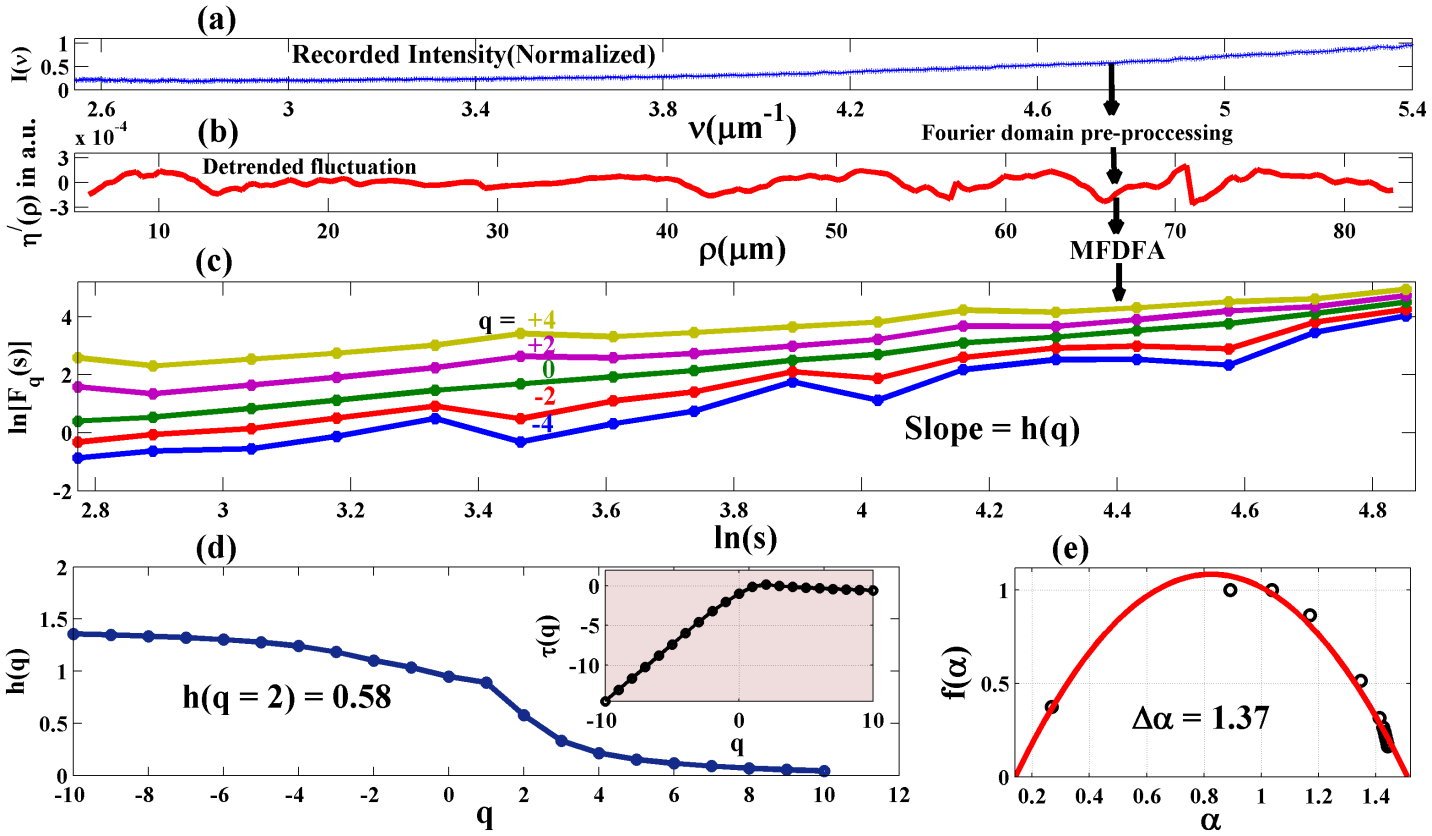
Results of the *inverse* analysis performed on the light scattering spectra recorded from a grade-1 precancer human cervical tissue slice (corresponding to Fig. 1). (a) The recorded light scattering intensity [I(ν) vs ν, 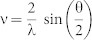, θ = 150°, λ = 360–740 nm, shown here]. (b) The representative index fluctuations with spatial scale ρ (*η*^/^(ρ)) extracted via Fourier domain pre-processing (using Eq. 6) on I(ν) (shown following polynomial detrending). (c), (d), (e): Results of the MFDFA *inverse* analysis on the detrended fluctuations *η*^/^(ρ). (c) The variation of log*F_q_(s)* vs log*s* for different moments *q*( = −4 to +4 shown here). Multifractality in *η*^/^(ρ) is evident from significant variations in the slopes with varying q. (d) The MFDFA-derived moment dependence of generalized Hurst exponent *h(q)* and classical scaling exponent *τ(q) (inset)*. (e) The resulting singularity spectrum *f(α)*. Close agreement in the behavior of *h(q)* and values for *h*(*q* = *2*) derived from this *inverse* analysis and that from the *forward* analysis of multifractality (Fig. 1d, e, f) suggests self-consistency.

Figure 3 displays the results of the *inverse* analysis performed on the light scattering spectra recorded (using the spectral light scattering measurement system, see Methods and Supplementary information) from a grade-1 dysplastic cervical tissue (corresponding to Fig. 1). Strong multifractality is once again evident from the large variations in the slopes of *logF_q_(s) vs logs* (Fig. 3c). The derived *h*(*q*) spectrum and the singularity spectrum *f(α)* (Fig. 3d and 3e) exhibits features similar to those obtained via *forward* analysis (Fig. 1e and 1f). Moreover, the derived h-value [*h*(*q* = *2*) * = * 0.58] is in good agreement with the *forward* analysis result [*h*(*q* = *2*) * = * 0.53]. This suggests self-consistency in *inverse* analysis of multifractality from tissue light scattering spectra. Note that even though the parameter *η*^/^(ρ), extracted via Fourier pre-processing, represents the index variations in a statistical sense over the probed tissue volume, it contains contributions of sub-micron level spatial index fluctuations (as evident from Fig. 3b). The multi-resolution capability of MFDFA allows one to further extract separately the contributing small scale (primarily via the negative moments *q*) and the large scale (via the positive moments *q*) fluctuations and quantify their statistical self-similar properties via *h(q)* (and *τ(q))* and *f(α)*^16^. The observed considerable variations of *h(q)* (*τ(q)*) for negative *q* -values (Fig. 3d) and the resulting width of *f(α)* (Fig. 3e) underscores the importance of the small scale index fluctuations (possibly originating from the micro-architecture of the fibrous network of connective tissue). It may be pertinent to discuss here about the statistical nature of the variation of the MFDFA-derived singularity spectrum *f(α)*. Note that *f(α)* contains relatively fewer data points for smaller values of *α.* This non-ideal nature of *f(α)* (as opposed to that expected for ideal multifractal fluctuation series) arises due to the strong contribution of the background Brownian type random fluctuations in the Fourier pre-processed light scattering data *η*^/^(ρ). Such background random fluctuations in turn originates from the associated randomness in the spatial index variation (as evident from Figure 1b, where the power spectrum of spatial index variation exhibit large background fluctuations in addition to the overall power law scaling behavior). Never-the-less, these results suggest that Fourier pre-processing of light scattering in Born approximation and its subsequent analysis through MFDFA captures and quantifies small spectral variations as signature of subtle (otherwise hidden) changes in the RI spatial distribution via the multifractal optical parameters. The possible interplay of the wavelength dependent tissue absorption on the light scattering inverse analysis method also worth a brief mention. Although, the biopsy tissue slices used in our study did not exhibit any significant spectral absorption features, in general, the light scattering spectra recorded from bulk tissues are expected to carry signature of absorption (primary candidates for absorptions in tissue in the visible wavelength region are the various forms of haemoglobins). Note that the signature of absorption typically manifests as sharp, localized (in the characteristic absorption bands) and distinct spectral features. Such regular trends in the spectral variation are expected to be removed via our pre-processing scheme of the light scattering spectral data (Fourier domain pre-processing employing Eq. 6 and subsequent detrending of the resulting fluctuations employing Eq. 1). Specifically, the de-trending process removes any regular trends (such as the ones resulting from distinct absorption spectral features) present in the Fourier pre-processed light scattering data *η*^/^(ρ). Moreover, one may also additionally employ methods such as polarization gating to reduce the signature of tissue absorption on light scattering spectra^13^,^18^.

**Figure 4:**
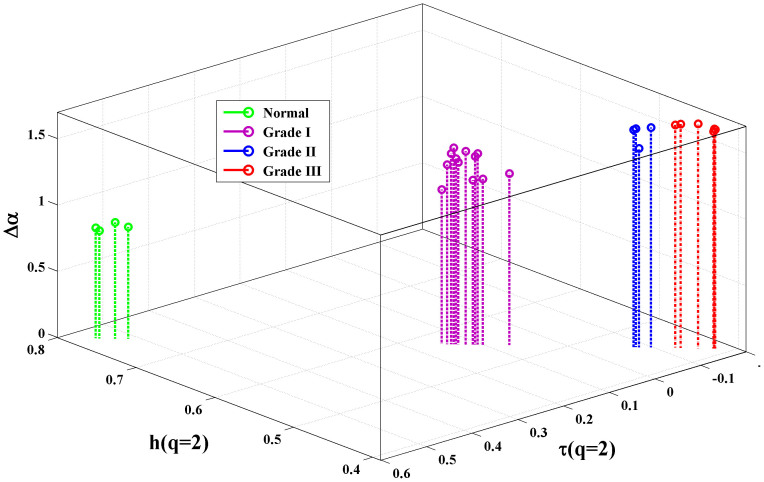
Discrimination of different grades of precancers based on the light scattering-derived *multifractal tissue optical properties*. Results from twenty two precancerous (Grade I - 12, Grade II -4 and Grade III -6) and four normal tissue sections are summarized. The multifractal parameters, Hurst exponent *h*(*q* = *2*), classical scaling exponent τ(*q* = *2*), and width of singularity spectrum Δα form the three axes. Higher grades of precancers are associated with increased anti-correlations of index fluctuations (increased roughness - reduction in *h*(*q* = *2*) and τ(*q* = *2*)) and stronger multifractality (increase in the value for Δα). These results provide some support those normal and precancerous tissues with different pathology grades can be discriminated using these derived multifractal parameters.

The newly derived *multifractal tissue optical properties* were investigated for discriminating different grades of cervical precancer. Twenty two precancerous and four normal connective tissue sections are analyzed in Figure 4. The observed trends are: *(i)* the values for *h*(*q* = *2*) or *τ*(*q* = *2*) are higher in normal tissues and decrease with increasing grades of precancer (e.g, *h*(*q* = *2*) = 0.77 ± 0.01, 0.56 ± 0.01 *and* 0.42 ± 0.01 *for normal, Grade I and Grade III tissues* respectively); *(ii)* the magnitudes of *Δα* are larger for higher grades of precancer (e.g. *Δα* = 0.84 ± 0.03, 1.36 ± 0.10 *and* 1.67 ± 0.02 *for normal, Grade I and Grade III tissues* respectively). Note that the limiting values for *h*(*q* = *2*) of unity and zero correspond to smooth Euclidean random field/marginal fractal (implying long range correlations) and a space-filling field/extreme fractal (anti correlated behavior) respectively. Reduction in the value for *h*(*q* = *2*) with increasing pathology thus likely indicates increasing tissue roughness, or effectively the predominance of index inhomogeneities having smaller dimensions. Connective tissue is comprised of collagen fiber network with complex organization of constituent micro-fibrils; statistical self-affinity of fibrous network may manifest as the observed multifractality in spatial variation of RI. Reduced *h*(*q* = *2*) may originate from volume fraction reduction of the fibrous network and/or fibers shortening/disorganization (both processes occurring with disease progression)^23^,^24^,^25^,^26^. Increased multifractality (larger value of *Δα*) at higher grades of precancer, on the other hand, may be a manifestation of the increased heterogeneity and the different scaling behavior of the small scale (possibly the constituent micro-fibril architecture in individual fibers) and the large scale (fibrous network) index fluctuations. This suggestion was initially tested in extracted Bovine collagen samples treated with 5% acetic acid (results not shown); this is known to degrade organized collagen fibrous structures by breaking cross-links^23^,^24^. Δα increased from ~1.4 to 1.8 after 30 minutes of acetic acid treatment. Note that we concentrate on connective tissue morphology (as opposed to the widely explored epithelial cell compartment) since progression of cancer involves altered interactions between epithelial cells and underlying connective tissue (stroma), and changes in stromal biology/morphology may precede and stimulate neoplastic progression in preinvasive disease^25^,^26^. The observed differences in the multifractal tissue scattering properties between normal and Grade I precancerous tissues thus points towards the ability of the method to quantify subtle morphological alterations in connective tissue as early signature of precancers.

In conclusion, a novel light scattering-based *inverse* method is developed, validated and explored for extraction and quantification of multifractality in the spatial RI fluctuations of biological tissues. Experimental validation of this *inverse* analysis method in synthetic multifractal scattering phantoms, and the agreement between the *forward* analysis on differential interference contrast image of tissue and *inverse* analysis from light scattering spectra, establishes self-consistency. The latter demonstrates that subtle changes in multifractal nature of tissue RI fluctuations can indeed be probed and quantified from light scattering spectra, despite numerous complexities associated with the wide range of spatial scaling of the index inhomogeneities, complex spatial correlations, and the resulting intertwined nature of their contribution to the tissue light scattering signal. The demonstrated ability to delineate multifractal properties may provide a valuable noninvasive tool for characterization of tissue and wide variety of other complex scattering media of non-biological origin. Observed differences in the *multifractal tissue optical properties* between different grades of precancers show considerable promise as potential biomarkers for early detection. The ability to probe and quantify subtle morphological and structural alterations associated with precancer using the elastic backscattering spectra bodes well for *in-vivo* deployment, because the backscattering geometry is clinically convenient and the measurement technique is fairly straight-forward. Exploiting the elastic backscattering spectra (singly or weakly scattered) recorded from superficial tissue depths, *in-vivo* applications of this approach can be realized with a suitably designed fiber optic probes and/or by other methods (e.g., polarization gating) to eliminate multiply scattered light^13^. Finally, the multifractal inverse analysis of light scattering represents a fundamentally novel approach with much potential, but its actual benefits for *in-vivo* detection of precancers remain to be rigorously evaluated. We are currently initiating such investigations in our laboratory.

## Methods

Histopathologically characterized (CIN or dysplasia grade I, II, and III) biopsy samples of human cervical tissues with normal counterparts were obtained from G. S. V. M. Medical College and Hospital in Kanpur, India (Patient age 35–60 years; n_total_ = 26, with n_grade I_ = 12, n_grade II_ = 4, n_grade III_ = 6; four biopsies from the normal counterparts, n_normal_ = 4). Standard histological preparation of the excised tissues involving fixation, dehydration, imbedding in wax, sectioning under a rotary microtome (thickness ~5 μm, lateral dimension ~4 mm × 6 mm), and subsequent de-waxing was employed. The control experiments were performed on the extracted Bovine collagen (Collagen from bovine achilles tendon, type I, C9879 SIGMA) samples. The collagen was treated with 5% (weight by volume) acetic acid and the light scattering spectra were recorded both before and after 30 minutes of treatment. Consent for the use of all the biopsy samples of human cervical tissues with normal counterparts in our study was obtained from the Ethical Committee, G. S. V. M. Medical College and Hospital, Kanpur, India. The methods were carried out in accordance with the approved guidelines.

A differential interference contrast (DIC) microscope (Olympus IX81, USA) was used to measure the spatial distribution of tissue refractive index (Figure 1a). Images were recorded with a CCD camera (ORCA-ERG, Hamamatsu, 1344 × 1024 pixels, pixel dimension 6.45 μm), at a magnification of 60×. The images were unfolded (pixel-wise) in one linear direction to obtain 1D fluctuation series, and were subsequently *forward* analyzed for multifractality employing Fourier and MFDFA methods. The elastic scattering spectra from the tissue sections were recorded using angle resolved spectral light scattering measurements (see Supplementary information). Briefly, light from a Xe-lamp (HPX-2000, Ocean Optics, USA) was collimated using a combination of lenses and illuminated the tissue sample at the centre of a goniometric arrangement (spot size ~1-mm-diameter). The sample-scattered light was collimated with lenses and was focused into a collecting fiber probe coupled to a spectrometer (USB4000FL, Ocean Optics, USA) for wavelength resolved signal detection. The spectra were recorded (360–800 nm) with a spectral resolution of 2.05 nm, for the angular range θ = 10–150°, at 10° intervals. The spectra recorded at backscattering angle θ = 150° was used for the *inverse* multifractal analysis (Figure 1c, Figure 3).

For validation of inverse analysis (Fig. 2), we employed Fourier optics experimental arrangement (Fig. 2a). Briefly, the 632.8 nm line of a He-Ne laser (HRR120-1, Thorlabs, USA) was used to seed the system. The beam was spatially filtered, collimated and then incident on a *transmissive* liquid crystal-based twisted nematic spatial light modulator (SLM, LC- 2002, Holoeye Photonics, Germany, 600 × 800 square pixels, pixel dimension 32 μm). The grey levels of the individual SLM pixels were modulated using user controlled inputs of either mono or multifractal fluctuation series. Fractional Brownian motion algorithm was used to generate monofractal fluctuation series with user controlled Hurst exponent (H) and multifractal fluctuation series with user controlled generalized Hurst exponent *h*(*q*) was generated using binomial multifractal model (see Supplementary information). Electrical addressing of the SLM pixels allowed the setting of the grey levels of each pixel according to the synthesized fluctuation series. This was achieved by setting the grey level of the central SLM pixel as the first element of the synthesized fluctuation series, the second element was used to set grey levels of all the neighboring pixels which made a square envelop, and so on. The SLM, acting as a synthetic mono/multifractal scattering (diffracting) object, was positioned at the front focal plane of the Fourier transforming lens (focal length *f*), and an EMCCD camera (iXon3-885, Andor Technology, UK, 1004 × 1002 square pixels, pixel dimension 8 μm) was at its back focal (Fourier) plane. The recorded intensity distribution at the CCD (x-y) plane represented the spatial frequency (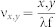) distribution of the mono/multifractal object (SLM).

## Author Contributions

N.G. designed and supervised this study; contributed in writing the manuscript, designing experimental strategies and theoretical models. N.D. and S.C. performed all the experiments (on tissues and control phantoms), analyzed data (both forward multifractal analysis on DIC images and inverse analysis on tissue light scattering data) contributed in writing the manuscript. A.V. and A.P. contributed in theoretical analysis/interpretation of data and in writing the manuscript. P.K.P. contributed in theoretical multifractal analysis, interpreting the data and writing the manuscript. S.K. contributed in the control validation studies on synthetic mono/multifractal scattering phantoms (both experiment and data analysis).

## Supplementary Material

Supplementary Information Tissue multifractality and Born approximation in analysis of light scattering: a novel approach for precancer detection
