# Supplementary material for: Tissue multifractality and Born approximation in analysis of light scattering: a novel approach for precancers detection
Source: Sci Rep. 2014 Aug 20;4:6129. doi: 10.1038/srep06129 (PMC4138517; doi:10.1038/srep06129)
Supplement: Supplementary Information — Tissue multifractality and Born approximation in analysis of light scattering: a novel approach for precancer detection [file srep06129-s1.pdf]

# *Supplementary Information*

## Tissue multifractality and Born approximation in analysis of light scattering: a novel approach for precancer detection

Nandan Das[1†], Subhasri Chatterjee[1†], Satish Kumar[1], Asima Pradhan[2], Prasanta Panigrahi[1], I. Alex Vitkin[3] and Nirmalya Ghosh[1,*]

[1]*Dept. of Physical Sciences, IISER- Kolkata, Mohanpur 741 252, Nadia, West Bengal, India*
[2]*Department of Physics, IIT Kanpur, Kanpur – 208016, India*
[3]*Department of Medical Biophysics and Radiation Oncology, University of Toronto, Canada M5G 2M9*

### A. Experimental System

***Spectral light scattering measurements system:***

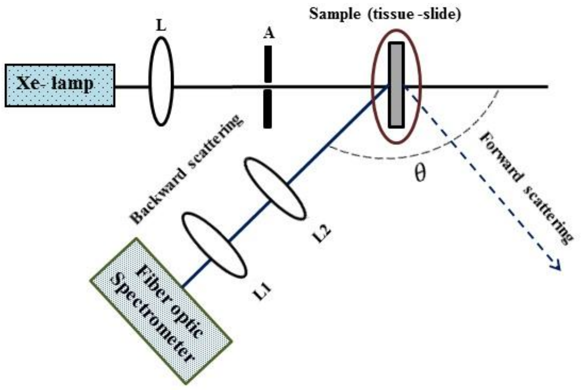


*Supplementary Figure 1: Schematic of the spectral light scattering measurement. Xe lamp: excitation source; A: aperture; L: collimating lens;* $L_1$ and $L_2$*: collecting lenses*

### B. Control validation experiments on synthetic mono / multifractal objects

#### *I. Realization of the mono/multifractal objects*

The standard fractional Brownian motion [1] (fbm) algorithm available in Matlab was used to generate monofractal fluctuation series with user controlled Hurst exponent (H). The multifractal fluctuation series was generated using binomial multifractal model. Briefly, the desired series was computed as [2, 3]

$$x_k = a^{n(k-1)}(1-a)^{n_{max}-n(k-1)} \tag{1}$$

Here, $N = 2^{n_{max}}$ with $k = 1, \dots, N$. n(k) is the number of digit equal to 1 in binary representation of the index k.

The generalized Hurst exponent $h(q)$is related to the user-defined parameter $a$ as

$$h(q) = \frac{1}{q} - \frac{ln\{a^q+(1-a)^q\}}{q\ ln2} \tag{2}$$

Electrical addressing of the SLM pixels allowed the setting of the grey levels of each pixel according to the synthesized fluctuation series. This was achieved by setting the grey level of the central SLM pixel as the first element of the synthesized fluctuation series, the second element was used to set grey levels of all the neighboring pixels which made a square envelop, and so on. Thus, full experimental control of the constituent fractal properties (Hurst exponent H for monofractal, generalized Hurst exponent $h(q)$ for multifractals) of the scattering object was available.

***II. Extraction of mono / multifractal properties from experimental light scattering data***

The Fourier power spectrum of any monofractal object assumes a power law behavior having a single exponent [2-4]

$$P(\nu) \approx \nu^{-\gamma} \tag{3}$$

Here, the power law exponent $\gamma = 2H + D_E$, $D_E$ is the Euclidian dimension ($D_E$ = 1, 2, 3 for one, two and three dimensional objects).

Since the scattered (diffracted) intensity from any object can be represented as the Fourier power spectrum of the object distribution, the experimentally recorded intensity distribution (either as a function of angle θ for a fixed wavelength λ, or as a function of λ for a fixed θ) from monofractal object should also exhibit power law scaling: $P(\nu) = \nu^{-(2H + D_E)}$. Here, ν is the spatial frequency = $\frac{2}{\lambda}\sin\left(\frac{\theta}{2}\right)$. The Hurst exponent *H* of the monofractal scattering (diffracting) objects were estimated from the slope of $\log P(\nu)$ vs $\log \nu$, and are listed in Supplementary Table 1. The estimated values for the Hurst exponent H are observed to be in close agreement with the controlled input.

| Controlled Input Hurst exponent (H) | Value of H determined from the theoretical Fourier power spectrum | Experimentally determined value of H |
|---|---|---|
| **0.3** | **0.26** | **0.34** |
| **0.4** | **0.44** | **0.41** |
| **0.5** | **0.48** | **0.52** |
| **0.6** | **0.56** | **0.57** |
| **0.7** | **0.65** | **0.72** |

*Supplementary Table 1: Results of the control experiment with monofractal objects (SLM pixels modulated through fluctuation series synthesized by FBM).*

The experimental intensity data obtained from the synthetic multifractal objects, on the other hand, exhibited multiple power law exponents at different spatial frequency ranges. These were therefore subjected to the *inverse* analysis method (MFDFA on Fourier pre-processed data). Representative results of such inverse analysis are presented in Figure 2 of the manuscript. Results obtained from various synthetic multifractal objects are summarized in Supplementary Table 2. The controlled generalized Hurst exponent *h(q=2)* input of the synthesized multifractal series, values for *h(q=2)* obtained by MFDFA analysis on the Fourier pre-processed theoretical power spectrum and experimental intensity data, are shown. Excellent agreement between the *a-priori* known values of *h(q=2)* and those obtained from MFDFA analysis on the Fourier pre-processed theoretical (power spectrum) and experimental intensity data provides evidence of self-consistency in *inverse* analysis of multifractality.

| Controlled input generalized Hurst exponent *h(q=2)* | *h(q=2)* from theoretical power spectrum (via Fourier pre-processing and MFDFA) | *h(q=2)* from experimental intensity data |
|---|---|---|
| **0.74** | **0.72** | **0.74** |
| **0.73** | **0.75** | **0.72** |
| **0.69** | **0.67** | **0.70** |
| **0.63** | **0.67** | **0.67** |
| **0.57** | **0.58** | **0.58** |

*Supplementary Table 2: Results of the control experiment with two dimensional multifractal objects (SLM pixels modulated through fluctuation series synthesized by binomial multifractal model).*

**References:**


1. Mandelbrot, Benoit B. and Ness, John W. Van. Fractional Brownian Motions, Fractional Noises and Applications. *SIAM Review* **10**, 422-437 (1968).
2. Feder, J. *Fractals* (Plenum Press**,** New York**,** 1988).
3. Kantelhardt, Jan. W. et al. Multifractal detrended fluctuation analysis of non-stationary time series. *Physica A* **316**, 87-114 (2002).
4. Peitgen,H.-O. Jüurgens, H. Saupe, D. Chaos and Fractals.(Springer**,** New York**,** 1992).
